# Beneficial Effect of Conversion to Belatacept in Kidney-Transplant Patients with a Low Glomerular-Filtration Rate

**DOI:** 10.1155/2014/190516

**Published:** 2014-05-18

**Authors:** Julie Belliere, Céline Guilbeau-Frugier, Arnaud Del Bello, Laure Esposito, Caroline Capuani, Isabelle Cardeau-Desangles, Lionel Rostaing, Nassim Kamar

**Affiliations:** ^1^Department of Nephrology and Organ Transplantation, CHU Rangueil, TSA 50032, 31059 Toulouse Cedex 9, France; ^2^Université Paul Sabatier, 31062 Toulouse, France; ^3^Department of Pathology, CHU Rangueil, 31059 Toulouse, France; ^4^INSERM U1043, IFR-BMT, CHU Purpan, 31059 Toulouse, France

## Abstract

Belatacept has been found to be efficient at preserving good kidney function in maintenance kidney-transplant patients. Herein, we report on the use of belatacept as a rescue therapy for two kidney-transplant patients presenting with severe adverse events after treatment with calcineurin inhibitors (CNIs) and mammalian target-of-rapamycin (mTOR) inhibitors. Two kidney-transplant patients developed severely impaired kidney function after receiving CNIs. The use of everolimus was associated with severe angioedema. Belatacept was then successfully used to improve kidney function in both cases, even though estimated glomerular-filtration rate before conversion was <20 mL/min. These case reports show that belatacept can be used as a rescue therapy, even if kidney function is very low in kidney-transplant patients who cannot tolerate CNIs and/or mTOR inhibitors.

## 1. Introduction

Interstitial fibrosis and tubular atrophy (IFTA) are two of the major causes of graft loss after kidney transplantation. Calcineurin inhibitors (CNIs) are well known to have nephrotoxic effects on the kidney allograft, leading to IFTA and graft loss [1]. Mammalian target-of-rapamycin (mTOR) inhibitors, such as sirolimus and everolimus, have been used in CNI-free regimens or in association with low-dose CNIs to reduce CNI dosage and, thus, nephrotoxicity [2]. However, although mTOR-based immunosuppression regimens can improve kidney function and reduce IFTA, their safety profile remains worrisome [2]. Indeed, their side effects are often unpredictable and lead to interruption of treatment in 40% of cases [3]. Hence, in some situations, patients can be intolerant and/or contraindicated to the large majority of immunosuppressive drugs. Consequently, preserving graft function and avoiding acute rejection then become a medical challenge.

Recently, belatacept (CTLA4-Ig) has been developed to block CD80/86 and thereby inhibit T-cell costimulation [4, 5]. Two phase-III trials have compared the efficacy and safety of belatacept to that of cyclosporine A in association with mycophenolate mofetil (MMF) and steroids in* de novo* kidney-transplant patients who had received a kidney allograft from standard- and extended-criteria donors. In belatacept-treated patients, although the incidence of acute rejection was slightly higher, long-term kidney function was significantly improved [6–9]. In addition, tolerance to belatacept was excellent.

Another phase-III study has assessed the effect of converting from CNIs (cyclosporine A or tacrolimus) to belatacept. Kidney-transplant patients, who had an estimated glomerular-filtration rate (using the MDRD equation) of between 35 and 75 mL/min, were randomized to be either maintained on CNIs or were converted to belatacept [10, 11]. The data collected over 3 years showed significantly better kidney function in patients converted to belatacept compared to those receiving CNIs (either tacrolimus or cyclosporine A) [12]. The effect of conversion from CNIs to belatacept, as a rescue therapy for kidney-transplant patients with a glomerular-filtration rate (GFR) of <35 mL/min, is unknown.

**Figure 1:**
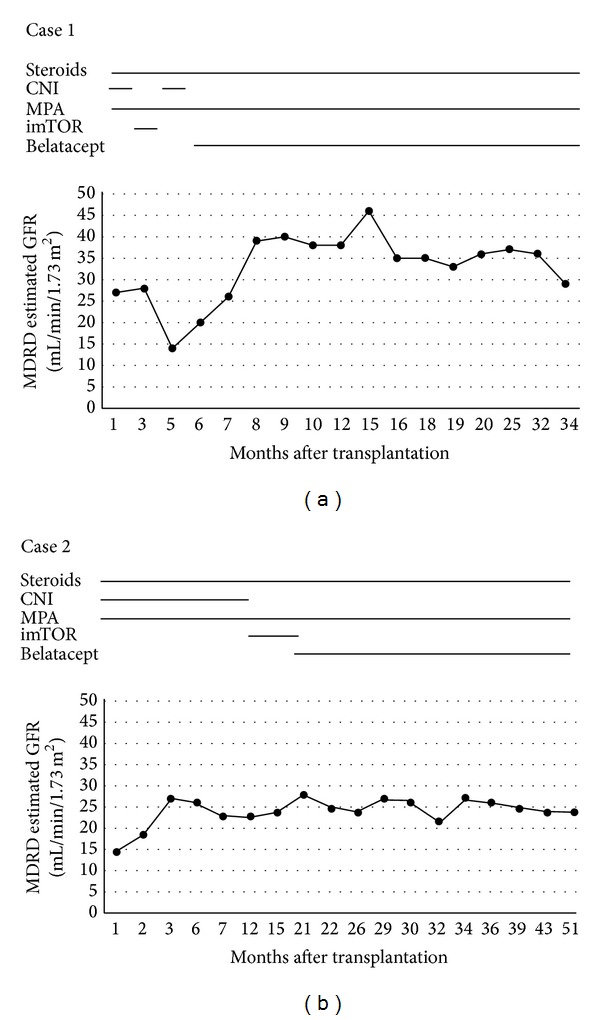
Kidney function. Glomerular-filtration rate (GFR) values were estimated with MDRD and reported for each case according to the time after transplantation. CNI: calcineurin inhibitors; MPA: mycophenolic acid; imTOR: mTOR (mammalian target of rapamycin) inhibitors.

Herein, we describe two kidney-transplant recipients with severe intolerance to CNIs and mTOR inhibitors who were successfully converted to belatacept. Glomerular-filtration rate (GFR) values are reported for each case in Figure 1.

## 2. Cases Reports

The patients' and donors' characteristics are presented in Table 1.

**Case 1**

A 52-year-old woman received a second kidney allograft for lupus nephritis and antiphospholipid antibody syndrome. The initial immunosuppressive therapy included basiliximab, tacrolimus, mycophenolic acid (MPA), and steroids. At one month after transplantation, because of persisting impaired kidney function (creatinine level 171 *μ*mol/L, eGFR of 27 mL/min/1.73 m²), a kidney biopsy was performed and showed ischemic tubular necrosis and signs of severe nephroangiosclerosis (t0 i0 g0 v0 ptc0 ah0 cg0 ci0 ct0 cv2 mm0, according to the Banff classification [13]), which was attributed to the donor, that is, a 77-year-old woman with a history of hypertension and who had died from a stroke. At that time, in order to avoid tacrolimus-induced nephrotoxicity, tacrolimus was replaced by everolimus. Kidney function remained unchanged. However, a switch back from everolimus to tacrolimus (target trough level 3–5 ng/mL) was done 2 months later because of severe angioedema. By five months after transplantation, the patient presented with heart failure, which was related to severe mitral-valve disease; thus, heart surgery was performed. After surgery, she developed acute kidney failure, which required dialysis for 6 days. Thereafter, her kidney function slightly improved but remained at ~400 *μ*mol/L (eGFR of 10 mL/min/1.73 m²). Hence, in order to avoid CNI-induced nephrotoxicity, tacrolimus was replaced by belatacept (5 mg/kg/month) at 6 months after transplantation, plus MPA (2 g/d) and steroids (5 mg/day). By 12 months after the switch, serum-creatinine level was 138 *μ*mol/L and eGFR was 35 mL/min/1.73 m².

At the last follow-up, that is, 29 months after the switch, serum-creatinine level and eGFR were, respectively, 162 *μ*mol/L and 29 mL/min/1.73 m². Proteinuria, which was 80 mg/g of creatinine before the switch, had decreased to 18 mg/g of creatinine at the last follow-up. Tolerance to the immunosuppressive treatment was excellent. No acute-rejection episode occurred and no donor-specific anti-HLA antibodies (DSAs) were detected using the Luminex single-antigen assay before or after the switch to belatacept.

**Case 2**

A 69-year-old man received a first kidney allograft for vascular nephropathy from an extended-criteria donor. The donor was a 74-year-old man with a history of diabetes and hypertension. The initial immunosuppressive therapy was based on basiliximab, tacrolimus, MPA, and steroids. After transplantation, his kidney function remained very poor. eGFR was 15, 26, and 23 mL/min/1.73 m² at 1, 6, and 12 months, respectively. A kidney biopsy, performed at 3 months posttransplantation, revealed signs of diabetic nephropathy and mild tubulopathy, but no rejection (t0 i0 g0 v0 ptc0 ah3 cg0 ci1 ct1 cv1 mm1, according to the Banff classification). At one year, tacrolimus was replaced by everolimus. Consequently, he presented with severe dyslipidemia and anemia, which required large doses of erythropoietin. In addition, steroid dose had to be reduced because of bilateral femoral osteonecrosis.

Another kidney biopsy was performed 6 months later, which showed grade-2 IFTA and severe vascular lesions (cv3, according to the Banff classification). Kidney function was altered; serum-creatinine level was 256 *μ*mol/L, eGFR was 22 mL/min/1.73 m², and proteinuria was 1.5 mg/g of creatinine. At that time, that is, 19 months after transplantation, everolimus was replaced by belatacept (5 mg/kg/month). MPA (2 g/d) and low-dose steroids (5 mg/d) were continued. At the last follow-up, that is, 33 months after the switch, serum-creatinine level was 224 *μ*mol/L, eGFR was 24 mL/min/1.73 m^2^, and proteinuria had decreased to 0.7 mg/g. Metabolic and hematological parameters were both improved. No DSAs were detected before or after the switch to belatacept.

## 3. Discussion

Within the last decade, the number of extended-criteria donors has increased. Consequently, for a large number of kidney-allograft recipients, the use of CNIs, which are known to have nephrotoxic effects, can be problematic. In* de novo* kidney-transplant patients who receive a kidney from an extended-criteria donor, the use of belatacept has been associated with significantly better kidney function at 5 years compared to patients that received cyclosporine A [6]. In maintenance kidney-transplant patients with preserved kidney function (eGFR between 35 and 75 mL/min), conversion from CNIs to belatacept significantly improved kidney function compared to those maintained on CNIs [10–12]. However, the effect of belatacept on kidney function in patients with impaired kidney function, that is, eGFR <35 mL/min, is unknown.

mTOR inhibitors have been used in conversion protocols to avoid CNI-induced nephrotoxicity [2]. However, late conversion from CNIs to mTOR inhibitors, when eGFR is <30 mL/min and/or when proteinuria is >0.5 mg/g of creatinine, does not prevent a decline in kidney function [14, 15]. In addition, mTOR inhibitors have several side effects that result in a high rate of treatment withdrawal, that is, 40% [3].

Herein, we have described two kidney-transplant recipients who were intolerant to both CNIs and mTOR inhibitors. The two kidney-transplant patients had severe impaired kidney function because of severe histological lesions related to the donor. The use of CNIs led to very low eGFR (<20 mL/min). The use of everolimus was associated with severe angioedema, requiring its withdrawal. Hence, belatacept was successfully used and led to improved kidney function in both cases, even though eGFR before conversion was <20 mL/min. Neither of the patients developed a serious adverse event, donor-specific antibodies, or posttransplant lymphoma disease.

In conclusion, these case reports highlight the fact that belatacept can be used as a rescue therapy, even if kidney function is very low, in kidney-transplant patients who cannot tolerate CNIs and/or mTOR inhibitors.

## Figures and Tables

**Table 1:** Donors' and recipients' characteristics.

	Case 1	Case 2
Donor		
Age (years)	77	60
Gender	W	W
Body mass index (kg/m^2^)	25.4	29
Cause of death	Hemorrhagic stroke	Ischemic stroke
Cardiac arrest	no	no
Serum creatinine level (µmol/L)	52	71
Proteinuria (g/L)	0.12	0.17
Estimated MDRD GFR (mL/min/1.73 m^2^)	100	74
Recipient		
Age (years)	52	69
Gender	W	M
Body mass index (kg/m^2^)	26.4	24.4
Cause of kidney disease	Lupus	Vascular nephropathy
Cold ischemia time (min)	765	690
Warm ischemia time (min)	45	60

W: woman; M: male; GFR: glomerular-filtration rate.
